# Dual Thrombophilic Mutations in Cerebral Venous Sinus Thrombosis: A Case Report Emphasizing Thrombophilia Screening and Indefinite Anticoagulation

**DOI:** 10.7759/cureus.91205

**Published:** 2025-08-28

**Authors:** Yu Sun, Weijing Liang, Liqiao Zhao, Yuli Hou, Xuan Chen

**Affiliations:** 1 Department of Neurology, Taiyuan Central Hospital, Taiyuan, CHN; 2 Department of Neurology, The First Hospital of Shanxi Medical University, Taiyuan, CHN

**Keywords:** anticoagulant therapy, cerebral venous sinus thrombosis (cvst), mthfr c677t, pai-1, thrombophilia screening

## Abstract

Cerebral venous sinus thrombosis (CVST), a rare stroke subtype, often involves inherited thrombophilias. We report a 40-year-old man presenting with progressive headache, blurred vision, and elevated intracranial pressure. Magnetic resonance venography confirmed CVST. Thrombophilia screening identified heterozygous methylenetetrahydrofolate reductase (MTHFR) C677T mutation and plasminogen activator inhibitor-1 (PAI-1) 4G/5G promoter polymorphism. These gene polymorphisms have been consistently associated with thrombosis development and prothrombotic states across studies, potentially indicating an underlying inherited thrombophilia in carriers. Initial therapy with anticoagulation (low molecular weight heparin/warfarin) and vitamin B supplementation resolved symptoms. Discontinuation of medication after 12 months resulted in thrombotic recurrence, which was reversed upon resumption of therapy. Serial monitoring showed sustained remission, normalized homocysteine, and therapeutic International Normalized Ratio (INR). This case highlights the synergistic thrombotic risk of coexisting MTHFR C677T and PAI-1 4G/5G variants in CVST, emphasizing the necessity of comprehensive thrombophilia screening to guide lifelong anticoagulation in hereditary hypercoagulable states. Adherence to indefinite therapy is critical to prevent recurrence in such patients.

## Introduction

Cerebral venous sinus thrombosis (CVST), accounting for 0.5%-3% of all strokes [1], is a rare cerebrovascular disorder with diverse etiologies. Established risk factors encompass inherited thrombophilias (e.g., protein C/S deficiencies, factor V Leiden mutation, plasminogen activator inhibitor-1 (PAI-1) polymorphisms, and prothrombin G20210A mutation), acquired hypercoagulable states (antiphospholipid syndrome, pregnancy/puerperium, hormonal therapies), infections, malignancy, and trauma [2]. This report describes a unique case of CVST in a young male patient harboring dual prothrombotic genetic variants, PAI-1 4G/5G promoter polymorphism and heterozygous methylenetetrahydrofolate reductase (MTHFR) C677T mutation, highlighting the imperative role of thrombophilia profiling in optimizing CVST management.

## Case presentation

A 40-year-old man presented with a five-day progressive headache (visual analog scale score: 6/10), localized bilaterally to the temporal and frontal regions, accompanied by dizziness, periorbital swelling, pain, nausea, and blurred vision. He denied thromboembolic family history, medication use, or traditional thrombotic risk factors. Neurological examination revealed blurred vision without any other neurological deficits.

**Figure 1:**
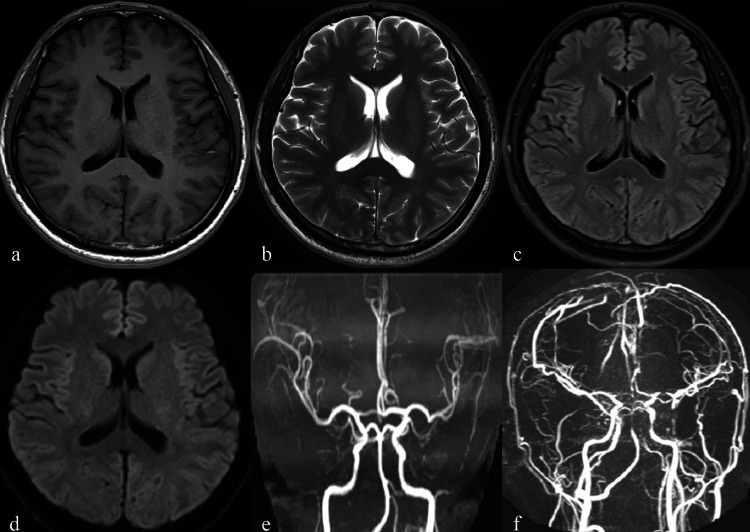
Images of the first onset. a-e: MRI and MRA showed unremarkable parenchymal and arterial structures. f: MRV demonstrated that the posterior part of the superior sagittal sinus, bilateral transverse sinuses, and sigmoid sinus were not visualized. The cerebral surface veins were significantly tortuous and thickened. MRI: magnetic resonance imaging; MRA: magnetic resonance angiography; MRV: magnetic resonance venography

Initial magnetic resonance imaging (MRI)/magnetic resonance angiography (MRA) showed unremarkable parenchymal and arterial structures, but magnetic resonance venography (MRV) demonstrated that the posterior part of the superior sagittal sinus, bilateral transverse sinuses, and sigmoid sinus were not visualized (Figure 1).

Additionally, the cerebral surface veins were significantly tortuous and thickened, suggesting a high likelihood of thrombosis. Laboratory findings included hyperhomocysteinemia (HHcy) (homocysteine (Hcy): 19.62 μmol/L; reference: 6-16 μmol/L). Lumbar puncture demonstrated significantly elevated intracranial pressure (>330 mm H_2_O) with subsequent cerebrospinal fluid (CSF) analysis revealing clear and colorless fluid. Biochemical analysis showed mildly increased protein concentration (0.59 g/L; reference: 0.2-0.45 g/L) while maintaining normal cellular components (white blood cell count <5/mm³, red blood cell count <10/mm³), glucose levels (2.8-4.4 mmol/L), and chloride concentrations (120-130 mmol/L). Based on the above examination and laboratory results, the patient was diagnosed with CVST. A comprehensive diagnostic workup was performed to exclude potential secondary etiologies, including acquired thrombophilic states, autoimmune diseases, and malignancies.

Thrombophilia screening identified a heterozygous MTHFR C677T mutation and PAI-1 4G/5G polymorphism, both associated with hypercoagulability. Anticoagulation with low-molecular-weight heparin (LMWH; 6000U Q12h) transitioning to warfarin (target International Normalized Ratio (INR): 2-3), mannitol for intracranial pressure, and vitamin B supplementation (folic acid, B12, B6) were initiated. Symptoms resolved after one month, and the patient was discharged on warfarin.

**Figure 2:**
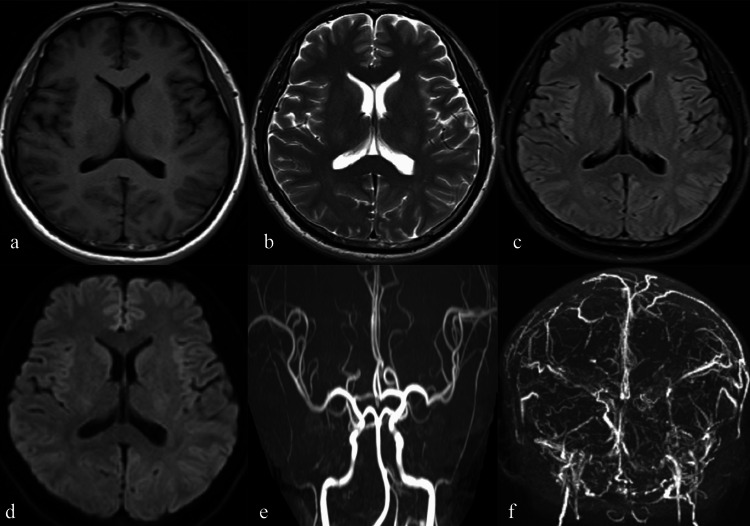
Images of disease recurrence. a-e: MRI and MRA showed unremarkable parenchymal and arterial structures. f: MRV showed thrombosis in bilateral transverse sinus, sigmoid sinus, sinus confluence, superior sagittal sinus and inferior sagittal sinus, and the veins on the brain surface were thickened and tortuous. MRI: magnetic resonance imaging; MRA: magnetic resonance angiography; MRV: magnetic resonance venography

After 12 months of therapeutic warfarin (INR 2-3) for cerebral venous thrombosis, the patient self-discontinued anticoagulation. Following four months of anticoagulation cessation, the patient developed recurrent symptoms characterized by progressive headache, left-sided paresthesia, and an emergent generalized tonic-clonic seizure. Repeat MRV confirmed extensive sinus thrombosis (Figure 2).

**Figure 3:**
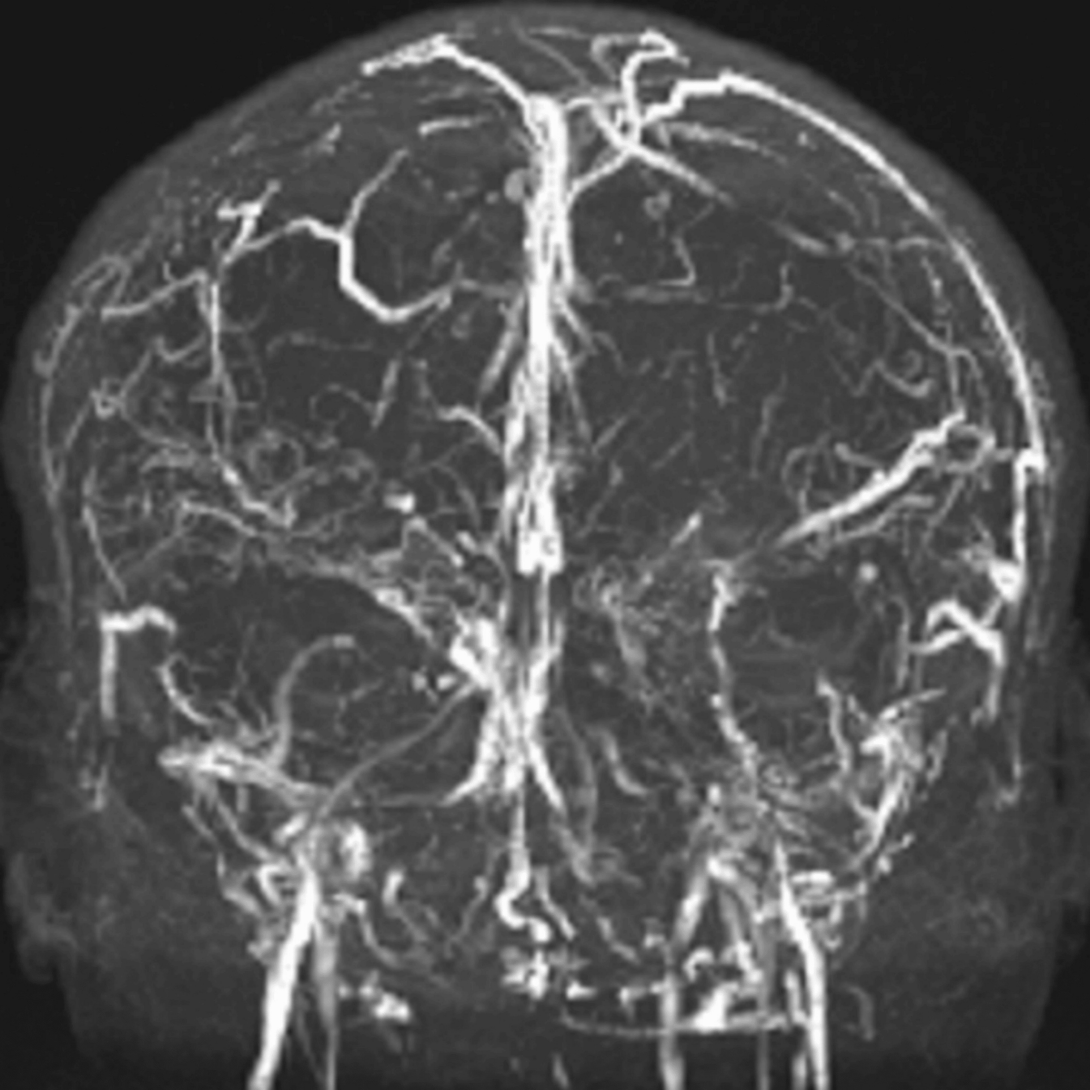
Follow-up image taken after a year. There is no significant change compared to the image from one year ago.

Hcy levels marginally increased (16.01 μmol/L; reference: 6-16 μmol/L), and CSF analysis showed elevated protein (0.68 g/L; reference: 0.2-0.45 g/L). Laboratory tests have again ruled out the possible causes of acquired hypercoagulable states, autoimmune disorders, and malignancies. Management included LMWH, levetiracetam for seizures, mannitol, and resumed warfarin (during the treatment period, the INR values were all within the standard range of 2-3). Symptoms improved, and no recurrence occurred during one-year follow-up with therapeutic INR (Figure 3).

## Discussion

CVST, accounting for 0.5%-3% of all strokes [1], is a rare cerebrovascular disorder with diverse etiologies. CVST manifests variably, ranging from headaches to seizures and focal neurological deficits [2]. This patient’s initial presentation (headache, blurred vision, elevated intracranial pressure) aligned with typical CVST features. CSF analysis excluded infectious etiologies. Neuroimaging confirmation was obtained through MRV, which conclusively demonstrated CVST.

The pathogenesis of CVST involves multifactorial interactions [2], with identified risk factors including acquired hypercoagulability, hereditary thrombophilia, hematologic cachexia, pharmacological triggers, autoimmune diseases, malignancies, and physical factors. Our patient is a previously healthy young man with no significant history of medication, family history of genetic disorders, or history of tobacco exposure, with normal comprehensive laboratory evaluations (including hematological analysis and immunological evaluation) except for mild HHcy. Elevated Hcy levels are an independent risk factor for the pathogenesis of CVST [3]. Experimental evidence suggests that HHcy enhances thrombosis through multiple pathways: oxidative stress induction, worsening of coagulopathy, vascular endothelial dysfunction, vascular smooth muscle hyperplasia, and intimal media hyperplasia [4]. While HHcy is often associated with aging, smoking, micronutrient deficiencies (folic acid, B12, B6), vegetarianism, and renal impairment, our patient does not exhibit these traditional susceptibilities. Therefore, a comprehensive thrombophile panel was implemented to evaluate FV Leiden (G1691A), prothrombin G20210A, MTHFR C677T polymorphisms, and functional assays of antithrombin III, protein C, protein S, and PAI-1. Genetic analysis revealed two notable findings: heterozygous MTHFR C677T mutations and 4G/5G polymorphisms in the PAI-1 promoter region. The MTHFR variant provides a plausible genetic basis for the observed HHcy, as it plays a key role in folate metabolism and homocysteine remethylation. Meanwhile, 4G/5G PAI-1 polymorphisms, known to enhance PAI-1 expression and impair fibrinolysis, suggest a synergistic prothrombotic mechanism. This combination of inherited thrombotic features establishes an innate susceptibility to thrombosis and may explain the CVST manifestations in this otherwise low-risk patient.

The MTHFR C677T polymorphism arises from cytosine-to-thymine substitution at nucleotide 677 [5], inducing alanine-to-valine substitution at codon 222 (A222V). This amino acid alteration disrupts the thermal stability of the enzyme, impairs its catalytic efficiency, and predisposes to moderate HHcy due to impaired folate-dependent homocysteine remethylation. As a key determinant of folate metabolism, the MTHFR C677T variant is strongly associated with genotypic variation in folate homeostasis: individuals carrying the TT or CT genotype exhibit significantly reduced serum folate levels and increased homocysteine concentrations compared to the CC homozygous genotype [6]. The evidence for its direct thrombotic effect is still controversial. Although a large Indian cohort study identified HHcy as an independent predictor of CVST, it found no significant association between MTHFR C677T polymorphisms and CVST incidence [7]. However, another study [8] suggested that this mutation may indirectly promote venous thrombotic events, including CVST, by increasing Hcy concentrations. In this setting, the patient's heterozygous CT genotype may result in mild but clinically relevant elevations in homocysteine, making it a modifiable prothrombotic risk factor in their congenital thrombophilia signature.

PAI-1 is a serine protease inhibitor synthesized primarily by endothelial and hepatocytes that acts as a key regulator of fibrinolysis by irreversibly inactivating tissue-type and urokinase-type plasminogen activators (tPA/uPA). Elevated PAI-1 expression or functional activity inhibits fibrinolytic capacity, thereby promoting thrombosis and recurrence. Genetic regulation produced by PAI-1 involves a site on chromosome 7q21.3 with a guanosine insertion/deletion polymorphism (4G/5G) in its promoter region. Compared to the 5G variant, the 4G allele confers transcriptional hyperactivity, leading to elevated plasma PAI-1 levels [9]. Although there is strong evidence that 4G/4G homozygous genotype is associated with an increased risk of venous thromboembolism and coronary artery disease, such as myocardial infarction, its association with CVST remains controversial [10]. Current studies suggest that the thrombotic effects of 4G/5G polymorphisms are context-dependent, mainly in the presence of coexisting congenital or acquired prothrombotic factors. In this patient, concurrent heterozygous MTHFR C677T mutations and PAI-1 4G/5G polymorphisms may produce synergistic prothrombotic interactions. MTHFR variant-induced HHcy may exacerbate endothelial dysfunction, while 4G allele-driven PAI-1 overexpression impairs clot resolution, a dual mechanism that enhances CVST development despite the absence of traditional risk factors.

Anticoagulation remains a cornerstone of CVST management, and the safety and efficacy of unfractionated heparin, LMWH, and oral anticoagulants have been widely validated [11]. Although endovascular intervention may be considered for clinical deterioration despite anticoagulation, contemporary evidence suggests that functional outcomes do not improve significantly compared to medical therapy alone and the risk of death may be increased [12]. In this setting, an anticoagulation strategy is implemented to initiate subcutaneous LMWH overlapping with dose-adjusted warfarin (target INR 2-3). Clinical improvement eliminates the need for invasive procedures. However, symptoms recur after voluntary discontinuation and resolve rapidly after anticoagulation is resumed. Synchronous HHcy management consists of daily supplementation with folic acid, B12, and B6. Afterwards, a 12-month follow-up was conducted. The patient took warfarin orally for a long time, and the INR value fluctuated within the standard range of 2-3, showing sustained clinical symptom relief and normalization of homocysteine levels (from 16.01 to 9.1 μmol/L; reference: 6-16 μmol/L), with no recurrent thrombosis observed. 

The optimal duration of anticoagulation therapy should be adjusted for the underlying cause. Patients with identifiable predisposing factors and resolved clinical manifestations may require a limited three-month course of treatment. Conversely, cases involving idiopathic hypercoagulable states require extended anticoagulation for 6-12 months [11]. It is important to note that hereditary thrombophilia requires permanent anticoagulation due to its persistent prothrombotic nature [13]. The thrombophilia panel of our patients revealed two clinically significant genetic polymorphisms: PAI-1 4G/5G and MTHFR C677T. These molecular markers confirm an inherited predisposition for thrombotic events. Following self-discontinuation of warfarin after 12 months of therapeutic anticoagulation (INR 2-3) with subsequent symptom recurrence, this case substantiates the necessity of indefinite anticoagulation therapy in patients with hereditary thrombophilia. In the present case, warfarin was selected for long-term anticoagulation. However, findings from the ACTION-CVT study indicate that in patients with CVST, direct oral anticoagulants (DOACs) demonstrate comparable rates of venous thromboembolism recurrence, mortality, and CVT recanalization to warfarin, while being associated with a lower risk of major hemorrhage. This suggests DOACs may achieve similar clinical and radiological outcomes with a superior safety profile. Nevertheless, these conclusions require further validation through large prospective cohort studies or randomized controlled trials. Additionally, the potential relationship between inherited thrombophilia and optimal anticoagulant selection remains undetermined and merits future investigation [14]. This case underscores the critical importance of comprehensive thrombophilia screening in CVST patients lacking significant risk factors. This diagnostic assessment serves a dual purpose: to inform evidence-based treatment decisions and to implement secondary prevention strategies for recurrent thrombosis.

## Conclusions

CVST necessitates a comprehensive etiological evaluation, involving detailed thrombophilia profiling to identify underlying causes and guide personalized therapeutic decisions. Early identification of specific genetic predispositions, such as mutations in the MTHFR or PAI-1 genes, critically informs the optimal duration of anticoagulation therapy and enhances secondary prevention strategies to mitigate recurrence risks. This particular case highlights the essential interplay of dual thrombophilic mutations in the pathogenesis of CVST, demonstrating how co-existing genetic factors amplify thrombotic tendencies, and it reinforces the crucial importance of sustained patient adherence to indefinite anticoagulation regimens in hereditary hypercoagulable states to prevent long-term complications.
